# Tree Diversity and Dynamics of the Forest of Seu Nico, Viçosa, Minas Gerais, Brazil

**DOI:** 10.3897/BDJ.3.e5425

**Published:** 2015-07-31

**Authors:** Markus Gastauer, Werner Leyh, João A.A. Meira-Neto

**Affiliations:** ‡ Federal University of Viçosa, Frutal, Brazil; § UFV, Viçosa, Brazil; | Federal University of Viçosa, Viçosa, Brazil

## Abstract

**Background:**

To understand future changes in community composition due to global changes, the knowledge about forest community dynamics is of crucial importance. To improve our understanding about processes and patterns involved in maintaining species rich Neotropical ecosystems, we provide here a dataset from the one hectare Forest of Seu Nico (FSN) Dynamics Plot from Southeastern Brazil.

**New information:**

We report diameter at breast height, basal area and height measurements of 2868 trees and treelets identified from two census spanning over a nine-year period. Furthermore, soil properties and understory light availability of all 100 10 x 10m subplots from the one hectare FSN Dynamics Plot during the second census are given.

## Introduction

Global changes such as habitat destruction, fragmentation and climate change threaten species richness and diversity of tropical forests (Wright 2010, Gastauer and Meira Neto 2013b, Magnago et al. 2014). To outline and understand their influences on tropical forest communities, long term monitoring studies, so-called community dynamics, are necessary (Losos and Leigh 2004, Wright 2005, Ernest et al. 2009, Laurance et al. 2014).

Among tropical forests, the Brazilian Atlantic Forest is one of the most diverse terrestrial ecosystems (Stehmann et al. 2009). Due to its high degree of endemism and endangered status it is considered a biodiversity hotspot (Myers et al. 2000). Once covering up to 1,500,000 km^2^ (Câmara 2005), only about 11 % of the original Brazilian Atlantic Forest remains, most of it as small secondary forest patches (Ribeiro et al. 2009). Species rich old-growth forests such as the Forest of Seu Nico (FSN) in the Viçosa municipality, Minas Gerais, Brazil are extremely rare and poorly studied (Campos et al. 2006, Gastauer and Meira Neto 2013a, Gastauer and Meira Neto 2013b). The aim of this data paper is to distribute dynamics data from the FSN Dynamics Plot in order to increase knowledge about community composition and maintenance in the Brazilian Atlantic Forest.

## Project description

### Title

Population and community dynamics of a Seasonal Semideciduous Primary Forest, Viçosa municipality, Minas Gerais, Brazil

### Study area description

**Figure 1.**
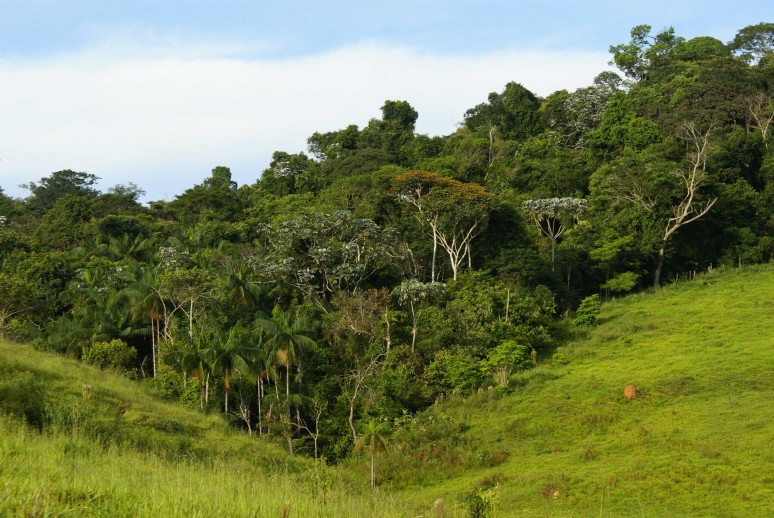
The Forest of Seu Nico (FSN) covers the bottom and the slopes of a small valley on the Bom Sucesso Farm in Viçosa, Minas Gerais, Brazil. Photograph by M. Gastauer from northeastern direction.

Located in Viçosa, Minas Gerais State, Brazil, the FSN is a forest fragment covering about 36 ha of a small valley (Fig. 1). The patch is surrounded by pastures, eucalypt plantations and secondary fragments such as the Reserva Florestal Mata do Paraíso (RFMP), that is, just as the FSN, used for training and research (http://www.def.ufv.br/infraEstruturaMataParaiso.php) by the nearby Federal University of Viçosa (UFV).

According to the Köppen system, the climate of Viçosa is characterized as mesothermic tropical highland climate with mild, rainy summers and cold, dry winters (Cwb) (Peel et al. 2007). Mean annual precipitation is arround 1200 mm, mean temperature is 19.4º C (Departamento Nacional de Meteorologia 1992). The predominant soils are deeply intemperished oxisols, but inceptisols are found on slopes and neosols in sedimentation areas. According to Veloso et al. (1991), the vegetation is characterized as Submontane Seasonal Semideciduous Forest.

The FSN has never been logged (Campos et al. 2006); despite selective wood and non-timber extraction, the fragment maintained primary forests characteristics such as high percentage of non-pioneer, animal-dispersed, understory and endemic species as well as high species richness and diversity (Gastauer 2012, Gastauer and Meira Neto 2013a, Gastauer and Meira Neto 2013b).

### Funding

JAAMN received a CNPq scholarship.

## Sampling methods

### Sampling description

**Figure 2.**
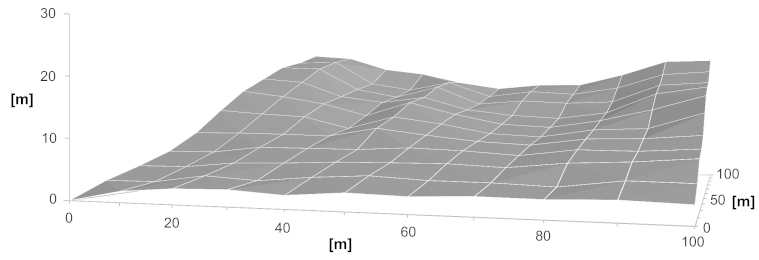
Croqui of the 100 x 100 m FSN Dynamics Plot (Suppl. material 1).

Within the FSN, a plot of 100 × 100 m was marked and divided into 100 subplots of 10 × 10 m. The plot is situated on the right side of the small valley covered by the FSN, showing northern exposure. The northern, lower part of the plot is flatter than its southern part (Fig. 2, Gastauer and Meira Neto 2013b).

During two censuses, all trees with a circumference at breast height greater than 10 cm were tagged and identified. This corresponds to a diameter at breast height (dbh) greater than 3.2 cm. During field campains, circumference of each individual fulfilling inclusion criterion was measured and the absolute height of trees was estimated. For multiple stem individuals, we calculated basal area at breast height for all shoots, summed these areas up and calculated from that the pooled circumference. dbh was computed from circumference assuming circular cross section of stems.

Specimens not recognized during field surveys were collected, deposited in the Herbarium of the Federal University of Viçosa (VIC) and identified with the help of material from the VIC, by consultation of specialists and/or literature. Species names were checked using the Taxonomic Name Resolution Service (TNRS) as proposed by Boyle et al. (2013); species classification follows Angiosperm Phylogeny Group III (2009).

During the second census, three soil samples were collected in each plot following a standardized protocol. For each sample four to five sampling points were defined, their systematic arrangement within each plot is shown in Gastauer and Meira Neto (2013b). At each sampling point, the organic layer was removed and a soil block of 10 × 10 × 20 cm (length × width × depth) was collected. Blocks from the same sample were mixed before 500 g were weighed, stored in a plastic bag and transported to the lab. Immediately after arrival at the lab, the soil samples were air-dried.

The following parameters were analyzed in the laboratories of the Soil Department of the Federal University of Viçosa: soil acidity as pH (extraction with water); the concentrations of phosphorus, potassium (both Mehlich 1 extraction), calcium, magnesium, and aluminum (extracted with 1 mol/L KCl); interchangeable bases; the effective cation exchange capacity as well as the cation exchange capacity at pH 7; and the saturation of bases, aluminum and remnant phosphorus.

Additionally, the understory light availability was analyzed by hemispherical photography during the second census. A digital camera (Nikon Coolpix 5700) was combined with an adapter and a fish-eye lens (Nikon FC-E9). For photography, the camera was mounted on a tripod. Within each plot, one photo was taken from plot´s center at an altitude of one meter above soil level. As direct light affects data interpretation and analysis, hemispherical photos were taken only when sky was perfectly overcast. Canopy openness, i.e. the percentage of open sky seen from beneath a forest canopy, as well as the amount of direct, diffuse and total solar radiation transmitted by the canopy were calculated by the software Gap Light Analyzer 2.0 (Frazer 1999).

## Geographic coverage

### Description

The FSN forest dynamics plot is situated in the Viçosa municipality, Minas Gerais, Brazil.

### Coordinates

-20.8 and Latitude; and -42.85 Longitude.

## Taxonomic coverage

### Description

Altogether 2868 trees belonging to 228 (morpho-)species from 54 families and 139 genera were sampled during both censuses (Table 1). Due to the lack of appropriate material (e.g., fruits or flowers) to provide a definite identification, 25 morphospecies remain partially or completely unidentified. 2143 individuals that were still present were resampled during the second census.

Although species richness and diversity declined from the first to the second census (Gastauer & Meira-Neto 2013), they are still outstanding for the region (Table 2, Lopes et al. 2002, Ferreira Júnior et al. 2007).

In terms of basal area, Moraceae is the most abundant family, while Fabaceae head the ranking in terms of species richness and Myrtaceae in number of individuals (Table 3). *Ficus* and *Pseudopiptadenia* are the most abundant genera in terms of basal area, while *Siparuna*, *Protium* and *Sorocea* are represented with the highest number of individuals and *Ocotea*, *Psychotria* and *Casearia* are most species-rich genera (Table 4). Three genera (*Himatanthus*, *Moldenhawera* and *Persea*) with one species each were registered only during the first census.

The largest tree in the sample is a *Ficus gomelleira* (dbh of 2.08 m), three further trees show a dbh lager than 1 m (*Ceiba speciosa*, *Sterculia curiosa* and *Astronium graveolens*) which explains the high rank of these species in terms of basal area (Table 5). The five species *Siparuna guianensis*, *Protium warmingiana*, *Sorocea bonplandii*, *Myrciaria floribunda* and *Euterpe edulis* count for more than 25% of all individuals (Table 5), on contrast, 34 species are represented by two individuals (i.e., Doubletons), and 62 species are Singletons, i.e. species with a single individual within our sample.

*Persea pyrifolia* (Lauraceae), *Himatanthus phagedaenicus* (Apocynaceae), *Croton hemiarhyreus* (Euphorbiaceae), *Jacaratia spinosa* (Caricaceae), *Moldenhawera* sp., *Inga* sp. (both Fabaceae), *Syagrus romazoffiana* (Arecaceae), *Lauraceae* sp. 3, *Monimiaceae* sp. and an unidentified species (Unidentified sp.3) were registered only during the first census and might be considered as pseudo-extinctions. On contrast, *Celtis iguanaea*, *Cybianthus fuscus*, *Vantanea obovata* and *Rubiaceae* sp. were registered only during the second census.

## Temporal coverage

### Notes

see Table 1

## Usage rights

### Use license

Creative Commons CCZero

### IP rights notes

This dataset can be freely used, provided it is cited.

## Data resources

### Data package title

Community dynamics of the Forest of Seu Nico, Viçosa municipality, Minas Gerais, Brazil

### Resource link

http://187.32.44.123/ipt/

### Alternative identifiers

http://187.32.44.123/ipt/resource.do?r=2015-06-04-gbif-ipt-unesco-ufv-seunico-fsn; http://www.gbif.org/dataset/3e95aa8e-a40c-4d34-ad11-7560484f1ff9

### Number of data sets

1

### Data set 1.

#### Data set name

dwca-2015-06-04-gbif-ipt-unesco-ufv-seunico-fsn.zip

#### Data format

Darwin Core Archive DwC-A

#### Number of columns

34

#### Description

2868 tree occurrences from two census within 100 plots of 10x10 m in the Forest of Seu Nico (FSN), Viçosa municipality, Minas Gerais, Brazil, including measurements of each tree as well as environmental data from all 100 plots. Dataset consists of seven independent files (Table 6)

**Data set 1.** 

Column label	Column description
id	Occurrence identifier
modified	Date of modification
language	The language of the resource
rights	Information about who can access the resource or an indication of its security status
rightsHolder	The organization owning and managing rights over the resource
bibliographicCitation	Bibliography citing this dataset
references	DOI of bibliography citing this dataset
datasetName	The name identifying the data set from which the record was derived
basisOfRecord	The specific nature of the data record
occurrenceID	Occurrence identifier
occurrenceRemarks	Comments or notes about the occurrence
eventDate	The date-time when the occurrence was recorded.
decimalLatitude	The geographic latitude in decimal degrees of the geographic position where occurrence was recorded
decimalLongitude	The geographic longitude in decimal degrees of the geographic position where occurrence was recorded
acceptedNameUsageID	Identifier for the name usage
parentNameUsageID	Identifier for the name usage
nameAccordingToID	Identifier for the source in which the specific taxon concept circumscription is defined or implied
scientificName	The full scientific name; when forming only part of an identification, name of lowest level taxonomic rank that was determined
acceptedNameUsage	Full name with authorship information of the sampled taxon
parentNameUsage	Full name of the direct, most proximate higher-rank parent taxon
nameAccordingTo	Reference to the source in which the specific taxon concept circumscription is defined or implied
higherClassification	List of taxa names terminating at the rank immediately superior to the taxon referenced in the taxon record, starting with the highest rank and separating the names for each rank with a semi-colon
kingdom	Full scientific name of the kingdom in which the taxon is classified
class	Full scientific name of the class in which the taxon is classified
order	Full scientific name of the order in which the taxon is classified
family	Full scientific name of the family in which the taxon is classified
genus	Full scientific name of the genus in which the taxon is classified
subgenus	Full scientific name of the subgenus in which the taxon is classified, when available
specificEpithet	Name of the species epithet of the scientificName
infraspecificEpithet	Name of the lowest or terminal infraspecific epithet of the scientificName
taxonRank	Taxonomic rank of the most specific name in the scientificName
scientificNameAuthorship	Authorship information for the scientificName
nomenclatureCode	The nomenclatural code under which the scientificName is constructed
taxonomicStatus	Status of the use of the scientificName as a label for a taxon linked to http://www.tropicos.org/

## Additional information

### Environmental data coverage

Minimum and maximum values of canopy openness differ by the factor 4.5, effective leaf area index show a twofold variation. Higher variation (factor 10) was observed for direct radiation than for diffuse radiation (factor 4), so that total radiation varies by factor 6 within the 100 subplots (Table 7). Nevertheless, influence of canopy openness or amount of radiation on tree species distribution has not yet been evaluated.

Minimum and maximum values of soil acidity, remnant phosphorus, cation exchange capacity at pH 7, nitrogen availability and organic material differ by the factor 1.4 to 2 among the 100 subplots; effective cation exchange capacity, potential soil acidity and phorphorus availability by factors between 2 and 3; potassium availability by factor 10 and saturation of bases, saturation of aluminium and availability of magnesium by factors up to 60. Aluminium saturation ranges from 0 to 95.1%, while aluminium and calcium availability range from 0 to 2.89 and 3.66 cmol^c^/dm^3^, respectively (Table 7). Nevertheless, soil variation explains only around 13% of species distribution within the 100 plots (Gastauer and Meira Neto 2013b).

As shown by Gastauer and Meira Neto (2013b), soil acidity increases with availability of potassium, calcium, magnesium as well as nitrogen. Furthermore, saturation of bases, effective cation exchange capacity, remnant phosphorus and nitrogen correlate positively with soil acidity, which decreases with increasing availability of aluminium. Phosphorus availability, on contrast, correlates positively with organic matter. Soil acidity does not correlate with percentage of canopy openess, but is correlated weakly with the amount of total solar radiation transmitted by the canopy (Gastauer 2012): The higher soil acidity, more light reaches understory. Aluminium availability, on the other hand, is significantly correlated with percentage of canopy openess and total solar radiation transmitted by the canopy. Percentage of canopy openess and total solar radiation transmitted by canopy is not correlated to number of individuals or species per plot, but pH correlates positively with per plot basal area and negatively with number of species and individuals per plot.

As outlined in Gastauer and Meira Neto (2013b), there is a soil gradient from south to north within the FSN dynamics plot. Southern subplots are more acidic, i.e., show lower soil acidity, have lower nitrogen, potassium and phosphorus contents, lower calcium and magnesium availability, a higher aluminium availability and lower saturation of bases as well as a lower effective cation exchange capacity than northern plots.

Due to correlations between soil properties and understory light availability, the percentage of canopy openess as well as the amount of solar radiation transmitted by canopy are higher in southern than in northern plots (Table 8)

### Description of the Darwin Core Archive containing dataset

Column labels and descriptions of further Darwin Core Archive files from both datasets are given at

Table 9 (measurementorfactplots.txt)

Table 10 (measurementorfactplants.txt)

Table 11 (resourcerelationship.txt)

Table 12​ (description.txt)

## Supplementary Material

Supplementary material 1 Elevation data of FSN Dynamics Plot Data type: X, Y and elevation values
Brief description: X, Y and elevation values of all vertices from 100 subplots from the one hectar FSN Dynamics Plot relative to starting point forming the northwestern vertex
File: oo_44956.txt Markus Gastauer, Werner Leyh, João A.A. Meira-Neto

Supplementary material 2 Seu Nico Community Dynamics Data type: Darwin Core Archive
Brief description: 2868 tree occurrences from two census within 100 plots of 10x10 m in the Forest of Seu Nico (FSN), Viçosa municipality, Minas Gerais, Brazil, including measurements of each tree as well as environmental data from all 100 plots. Dataset consists of seven independent files
File: oo_44941.zip Markus Gastauer, Werner Leyh, João Augusto Alves Meira-Neto

## Figures and Tables

**Table 1.** FSN plot census history.

**Census**	**Dates**	**Number of trees**	**Number ​of species**	**Number of trees ≥ 10 cm**	**Number of species (≥ 10 cm dbh)**
1	October 2000-March 2001	2482	224	762	154
2	December 2009-February 2010	2529	218	721	154

**Table 2.** FSN diversity and species richness summary tally for the second census. N is number of individual trees, S is number of species, G is number of genera, F is number of families, H‘ is Shannon-Wiener diversity index using log_10_, and α is Fisher´s α. Basal area (BA) includes all multiple stems for each individual.

**Size Class [cm dbh]**	**BA [m^2^]**	**N**	**S**	**G**	**F**	**H‘**	**α ±DP**
≥ 3.2	40.185	2529	218	136	54	4.36	57.19 ±2.16
≥ 10	35.663	762	154	107	38	4.39	58.21 ±3.41
≥ 30	20.535	108	51	46	23	3.52	37.75 ±6.06
≥ 60	9.782	11	7	7	6	-	-

**Table 3.** FSN rankings by family, data from the second census. BA is basal area and includes all multiple stems for each individual, N is number of individuals and S is number of species.

**Rank**	**Family**	**BA**	**% BA**	**% N**	**Family**	**N**	**% N**	**Family**	**S**
1	Moraceae	6.40	15.92	10.28	Myrtaceae	368	14.55	Fabaceae	21
2	Fabaceae	5.65	14.05	6.29	Siparunaceae	262	10.36	Myrtaceae	19
3	Malvaceae	3.76	9.36	1.11	Moraceae	260	10.28	Rubiaceae	16
4	Myristicaceae	2.78	6.91	3.80	Lauraceae	221	8.74	Lauraceae	15
5	Annonaceae	2.69	6.70	3.56	Rubiaceae	162	6.41	Euphorbiaceae	11
6	Lauraceae	2.40	5.97	8.74	Fabaceae	159	6.29	Moraceae	10
7	Burseraceae	1.89	4.70	5.34	Burseraceae	135	5.34	Meliaceae	10
8	Myrtaceae	1.56	3.88	14.55	Meliaceae	107	4.23	Salicaceae	9
9	Urticaceae	1.49	3.72	1.74	Myristicaceae	96	3.80	Annonaceae	8
10	Rubiaceae	1.42	3.54	6.41	Arecaceae	95	3.76	Sapotaceae	6
11	Meliaceae	1.39	3.46	4.23	Annonaceae	90	3.56	Melastomataceae	6
12	Salicaceae	1.39	3.46	2.29	Sapotaceae	82	3.24	Malvaceae	4
13	Anacardiaceae	1.27	3.17	1.30	Salicaceae	58	2.29	Urticaceae	4
14	Arecaceae	0.77	1.92	3.76	Urticaceae	44	1.74	Anacardiaceae	4
15	Sapotaceae	0.67	1.66	3.24	Anacardiaceae	33	1.30	Celastraceae	4
16	Siparunaceae	0.60	1.49	10.36	Celastraceae	32	1.27	Nyctaginaceae	4
17	Euphorbiaceae	0.48	1.19	1.11	Malvaceae	28	1.11	Apocynaceae	4
18	Sapindaceae	0.45	1.12	0.63	Euphorbiaceae	28	1.11	Solanaceae	4
19	Chrysobalanaceae	0.33	0.82	0.59	Nyctaginaceae	28	1.11	Burseraceae	3
20	Celastraceae	0.32	0.81	1.27	Olacaceae	27	1.07	Sapindaceae	3
21	Olacaceae	0.26	0.65	1.07	Clusiaceae	25	0.99	Bignoniaceae	3
22	Nyctaginaceae	0.22	0.55	1.11	Melastomataceae	21	0.83	Clusiaceae	3
23	Bignoniaceae	0.22	0.54	0.51	Sapindaceae	16	0.63	Primulaceae	3
24	Boraginaceae	0.21	0.53	0.16	Chrysobalanaceae	15	0.59	Myristicaceae	2
25	Phyllanthaceae	0.19	0.46	0.16	Bignoniaceae	13	0.51	Arecaceae	2
26	Clusiaceae	0.14	0.34	0.99	Apocynaceae	13	0.51	Siparunaceae	2
27	Lythraceae	0.14	0.34	0.32	Piperaceae	11	0.43	Chrysobalanaceae	2
28	Apocynaceae	0.13	0.33	0.51	Lythraceae	8	0.32	Olacaceae	2
29	Melastomataceae	0.13	0.33	0.83	Ochnaceae	8	0.32	Phyllanthaceae	2
30	Rutaceae	0.13	0.32	0.12	Sabiaceae	8	0.32	Rutaceae	2
31	Araliaceae	0.10	0.25	0.28	Araliaceae	7	0.28	Araliaceae	2
32	Rhamnaceae	0.09	0.22	0.04	Lacistemataceae	7	0.28	Lecythidaceae	2
33	Vochysiaceae	0.09	0.22	0.04	Primulaceae	7	0.28	Piperaceae	2
34	Lacistemataceae	0.08	0.19	0.28	Solanaceae	6	0.24	Erythroxylaceae	2
35	Ochnaceae	0.06	0.15	0.32	Achariaceae	6	0.24	Boraginaceae	1
36	Unidentified	0.06	0.14	0.08	Monimiaceae	5	0.20	Lythraceae	1
37	Lecythidaceae	0.04	0.10	0.16	Boraginaceae	4	0.16	Rhamnaceae	1
38	Calophyllaceae	0.03	0.08	0.12	Phyllanthaceae	4	0.16	Vochysiaceae	1
39	Solanaceae	0.02	0.06	0.24	Lecythidaceae	4	0.16	Lacistemataceae	1
40	Primulaceae	0.02	0.05	0.28	Cardiopteridaceae	4	0.16	Ochnaceae	1
41	Asteraceae	0.02	0.05	0.04	Rutaceae	3	0.12	Calophyllaceae	1
42	Achariaceae	0.02	0.04	0.24	Calophyllaceae	3	0.12	Asteraceae	1
43	Piperaceae	0.01	0.04	0.43	Unidentified	2	0.08	Achariaceae	1
44	Sabiaceae	0.01	0.04	0.32	Erythroxylaceae	2	0.08	Sabiaceae	1
45	Erythroxylaceae	0.01	0.03	0.08	Aquifoliaceae	2	0.08	Cardiopteridaceae	1
46	Cardiopteridaceae	0.01	0.02	0.16	Rhamnaceae	1	0.04	Monimiaceae	1
47	Monimiaceae	0.01	0.02	0.20	Vochysiaceae	1	0.04	Aquifoliaceae	1
48	Aquifoliaceae	0.01	0.02	0.08	Asteraceae	1	0.04	Elaeocarpaceae	1
49	Elaeocarpaceae	0.01	0.02	0.04	Elaeocarpaceae	1	0.04	Rosaceae	1
50	Rosaceae	0.00	0.01	0.04	Rosaceae	1	0.04	Caricaceae	1
51	Caricaceae	0.00	0.01	0.04	Caricaceae	1	0.04	Combretaceae	1
52	Combretaceae	0.00	0.01	0.04	Combretaceae	1	0.04	Cannabaceae	1
53	Cannabaceae	0.00	0.00	0.04	Cannabaceae	1	0.04	Humiriaceae	1
54	Humiriaceae	0.00	0.00	0.04	Humiriaceae	1	0.04	Oleaceae	1
55	Oleaceae	0.00	0.00	0.04	Oleaceae	1	0.04	Unidentified	2

**Table 4.** FSN ranking by genus, data from the second census. BA is basal area, N is number of individuals and S is number of species.

**Rank**	**Genus**	**BA**	**% BA**	**% N**	**Genus**	**N**	**% N**	**Genus**	**S**
1	*Ficus* (Moraceae)	3.43	8.54	0.12	*Siparuna* (Siparunaceae)	262	10.36	*Ocotea* (Lauraceae)	6
2	*Pseudopiptadenia* (Fabaceae)	3.08	7.67	1.78	*Protium* (Burseraceae)	129	5.10	*Psychotria* (Rubiaceae)	6
3	*Virola* (Myristicaceae)	2.78	6.91	3.80	*Sorocea* (Moraceae)	126	4.98	*Casearia* (Salicaceae)	5
4	*Guatteria* (Annonaceae)	1.97	4.91	2.93	*Ocotea* (Lauraceae)	103	4.07	*Miconia* (Melastomataceae)	5
5	*Sterculia* (Malvaceae)	1.84	4.58	0.47	*Myrciaria* (Myrtaceae)	101	4.00	*Myrcia* (Myrtaceae)	5
6	*Ocotea* (Lauraceae)	1.73	4.30	4.07	*Virola* (Myristicaceae)	96	3.80	*Chrysophyllum* (Sapotaceae)	4
7	*Sorocea* (Moraceae)	1.60	3.97	4.98	*Euterpe* (Arecaceae)	90	3.56	*Eugenia* (Myrtaceae)	4
8	*Protium* (Burseraceae)	1.37	3.42	5.10	*Marlierea* (Myrtaceae)	81	3.20	*Guarea* (Meliaceae)	4
9	*Ceiba* (Malvaceae)	1.37	3.41	0.04	*Trichilia* (Meliaceae)	76	3.01	*Guatteria* (Annonaceae)	4
10	*Astronium* (Anacardiaceae)	1.06	2.63	0.67	*Guatteria* (Annonaceae)	74	2.93	*Inga* (Fabaceae)	4
11	*Casearia* (Salicaceae)	1.03	2.56	1.90	*Helicostylis* (Moraceae)	73	2.89	*Trichilia* (Meliaceae)	4
12	*Trichilia* (Meliaceae)	0.74	1.84	3.01	*Myrcia* (Myrtaceae)	71	2.81	*Aspidosperma* (Apocynaceae)	3
13	*Bathysa* (Rubiaceae)	0.68	1.70	2.65	*Bathysa* (Rubiaceae)	67	2.65	*Ficus* (Moraceae)	3
14	*Pourouma* (Urticaceae)	0.66	1.64	1.23	*Eugenia* (Myrtaceae)	64	2.53	*Machaerium* (Fabaceae)	3
15	*Helicostylis* (Moraceae)	0.64	1.60	2.89	*Phyllostemonodaphne* (Lauraceae)	59	2.33	*Marlierea* (Myrtaceae)	3
16	*Euterpe* (Arecaceae)	0.64	1.59	3.56	*Chrysophyllum* (Sapotaceae)	56	2.22	*Maytenus* (Celastraceae)	3
17	*Inga* (Fabaceae)	0.63	1.57	1.34	*Brosimum* (Moraceae)	49	1.94	*Alchornea* (Euphorbiaceae)	2
18	*Annona* (Annonaceae)	0.60	1.50	0.40	*Casearia* (Salicaceae)	48	1.90	*Annona* (Annonaceae)	2
19	*Siparuna* (Siparunaceae)	0.60	1.49	10.36	*Pseudopiptadenia* (Fabaceae)	45	1.78	*Astronium* (Anacardiaceae)	2
20	*Brosimum* (Moraceae)	0.59	1.48	1.94	*Inga* (Fabaceae)	34	1.34	*Bathysa* (Rubiaceae)	2
21	*Guarea* (Meliaceae)	0.59	1.47	1.11	*Alseis* (Rubiaceae)	31	1.23	*Cariniana* (Lecythidaceae)	2
22	*Trattinnickia* (Burseraceae)	0.51	1.28	0.24	*Pourouma* (Urticaceae)	31	1.23	*Cestrum* (Solanaceae)	2
23	*Eriotheca* (Malvaceae)	0.50	1.25	0.51	*Maytenus* (Celastraceae)	29	1.15	*Coussapoa* (Urticaceae)	2
24	*Coussapoa* (Urticaceae)	0.49	1.21	0.16	*Guarea* (Meliaceae)	28	1.11	*Erythroxylum* (Erythroxylaceae)	2
25	*Chrysophyllum* (Sapotaceae)	0.48	1.19	2.22	*Neomitranthes* (Myrtaceae)	26	1.03	*Guapira* (Nyctaginaceae)	2
26	*Piptadenia* (Fabaceae)	0.38	0.94	0.24	*Pouteria* (Sapotaceae)	24	0.95	*Nectandra* (Lauraceae)	2
27	*Cecropia* (Urticaceae)	0.35	0.86	0.36	*Urbanodendron* (Lauraceae)	23	0.91	*Piper* (Piperaceae)	2
28	*Matayba* (Sapindaceae)	0.35	0.86	0.47	*Aniba* (Lauraceae)	22	0.87	*Protium* (Burseraceae)	2
29	*Myrciaria* (Myrtaceae)	0.33	0.83	4.00	*Calyptranthes* (Myrtaceae)	20	0.79	*Siparuna* (Siparunaceae)	2
30	*Eugenia* (Myrtaceae)	0.33	0.82	2.53	*Psychotria* (Rubiaceae)	20	0.79	*Sorocea* (Moraceae)	2
31	*Myrcia* (Myrtaceae)	0.30	0.74	2.81	*Tetrastylidium* (Olacaceae)	20	0.79	*Swartzia* (Fabaceae)	2
32	*Phyllostemonodaphne* (Lauraceae)	0.30	0.74	2.33	*Amaioua* (Rubiaceae)	18	0.71	*Tapirira* (Anacardiaceae)	2
33	*Marlierea* (Myrtaceae)	0.27	0.68	3.20	*Apuleia* (Fabaceae)	18	0.71	*Virola* (Myristicaceae)	2
34	*Machaerium* (Fabaceae)	0.26	0.64	0.40	*Astronium* (Anacardiaceae)	17	0.67	*Xylopia* (Annonaceae)	2
35	*Hirtella* (Chrysobalanaceae)	0.24	0.59	0.51	*Swartzia* (Fabaceae)	16	0.63	*Allophylus* (Sapindaceae)	1
36	*Tetrastylidium* (Olacaceae)	0.23	0.57	0.79	*Tapirira* (Anacardiaceae)	16	0.63	*Alseis* (Rubiaceae)	1
37	*Tapirira* (Anacardiaceae)	0.22	0.54	0.63	*Copaifera* (Fabaceae)	15	0.59	*Amaioua* (Rubiaceae)	1
38	*Maytenus* (Celastraceae)	0.22	0.54	1.15	*Guapira* (Nyctaginaceae)	15	0.59	*Andira* (Fabaceae)	1
39	*Cordia* (Boraginaceae)	0.21	0.53	0.16	*Mouriri* (Melastomataceae)	14	0.55	*Aniba* (Lauraceae)	1
40	*Jacaranda* (Bignoniaceae)	0.21	0.52	0.40	*Eriotheca* (Malvaceae)	13	0.51	*Aparisthmium* (Euphorbiaceae)	1
41	*Macrothumia* (Salicaceae)	0.19	0.48	0.04	*Hirtella* (Chrysobalanaceae)	13	0.51	*Apuleia* (Fabaceae)	1
42	*Amaioua* (Rubiaceae)	0.18	0.45	0.71	*Ixora* (Rubiaceae)	12	0.47	*Astrocaryum* (Arecaceae)	1
43	*Ormosia* (Fabaceae)	0.18	0.44	0.08	*Matayba* (Sapindaceae)	12	0.47	*Brosimum* (Moraceae)	1
44	*Ixora* (Rubiaceae)	0.18	0.44	0.47	*Sterculia* (Malvaceae)	12	0.47	*Brunfelsia* (Solanaceae)	1
45	*Copaifera* (Fabaceae)	0.17	0.41	0.59	*Aspidosperma* (Apocynaceae)	11	0.44	*Cabralea* (Meliaceae)	1
46	*Neomitranthes* (Myrtaceae)	0.17	0.41	1.03	*Piper* (Piperaceae)	11	0.44	*Calyptranthes* (Myrtaceae)	1
47	*Hymenaea* (Fabaceae)	0.16	0.39	0.08	*Annona* (Annonaceae)	10	0.40	*Campomanesia* (Myrtaceae)	1
48	*Andira* (Fabaceae)	0.16	0.39	0.08	*Jacaranda* (Bignoniaceae)	10	0.40	*Carpotroche* (Achariaceae)	1
49	*Alseis* (Rubiaceae)	0.15	0.37	1.23	*Machaerium* (Fabaceae)	10	0.40	*Cecropia* (Urticaceae)	1
50	*Sapium* (Euphorbiaceae)	0.14	0.35	0.16	*Nectandra* (Lauraceae)	10	0.40	*Cedrela* (Meliaceae)	1
51	*Lafoensia* (Lythraceae)	0.14	0.34	0.32	*Pisonia* (Nyctaginaceae)	10	0.40	*Ceiba* (Malvaceae)	1
52	*Astrocaryum* (Arecaceae)	0.13	0.33	0.20	*Tovomitopsis* (Clusiaceae)	10	0.40	*Celtis* (Cannabaceae)	1
53	*Aniba* (Lauraceae)	0.12	0.31	0.87	*Cecropia* (Urticaceae)	9	0.36	*Cinnamomum* (Lauraceae)	1
54	*Nectandra* (Lauraceae)	0.12	0.31	0.40	*Garcinia* (Clusiaceae)	8	0.32	*Citronella* (Cardiopteridaceae)	1
55	*Zanthoxylum* (Rutaceae)	0.12	0.30	0.08	*Lafoensia* (Lythraceae)	8	0.32	*Clarisia* (Moraceae)	1
56	*Calyptranthes* (Myrtaceae)	0.12	0.30	0.79	*Meliosma* (Sabiaceae)	8	0.32	*Colubrina* (Rhamnaceae)	1
57	*Xylopia* (Annonaceae)	0.12	0.29	0.24	*Ouratea* (Ochnaceae)	8	0.32	*Copaifera* (Fabaceae)	1
58	*Psychotria* (Rubiaceae)	0.12	0.29	0.79	*Heisteria* (Olacaceae)	7	0.28	*Cordia* (Boraginaceae)	1
59	*Pisonia* (Nyctaginaceae)	0.12	0.29	0.40	*Lacistema* (Lacistemataceae)	7	0.28	*Croton* (Euphorbiaceae)	1
60	*Dalbergia* (Fabaceae)	0.11	0.28	0.12	*Miconia* (Melastomataceae)	7	0.28	*Cryptocarya* (Lauraceae)	1
61	*Croton* (Euphorbiaceae)	0.11	0.28	0.16	*Tovomita* (Clusiaceae)	7	0.28	*Cupania* (Sapindaceae)	1
62	*Salacia* (Celastraceae)	0.11	0.27	0.12	*Carpotroche* (Achariaceae)	6	0.24	*Cybianthus* (Primulaceae)	1
63	*Hieronyma* (Phyllanthaceae)	0.10	0.25	0.08	*Piptadenia* (Fabaceae)	6	0.24	*Dalbergia* (Fabaceae)	1
64	*Pouteria* (Sapotaceae)	0.10	0.24	0.95	*Rudgea* (Rubiaceae)	6	0.24	*Dendropanax* (Araliaceae)	1
65	*Schefflera* (Araliaceae)	0.10	0.24	0.24	*Schefflera* (Araliaceae)	6	0.24	*Eriotheca* (Malvaceae)	1
66	*Licania* (Chrysobalanaceae)	0.09	0.23	0.08	*Trattinnickia* (Burseraceae)	6	0.24	*Euterpe* (Arecaceae)	1
67	*Swartzia* (Fabaceae)	0.09	0.23	0.63	*Xylopia* (Annonaceae)	6	0.24	*Garcinia* (Clusiaceae)	1
68	*Pradosia* (Sapotaceae)	0.09	0.23	0.08	*Alchornea* (Euphorbiaceae)	5	0.20	*Genipa* (Rubiaceae)	1
69	*Guapira* (Nyctaginaceae)	0.09	0.22	0.59	*Astrocaryum* (Arecaceae)	5	0.20	*Guettarda* (Rubiaceae)	1
70	*Colubrina* (Rhamnaceae)	0.09	0.22	0.04	*Mollinedia* (Monimiaceae)	5	0.20	*Handroanthus* (Bignoniaceae)	1
71	*Qualea* (Vochysiaceae)	0.09	0.22	0.04	*Myrsine* (Primulaceae)	5	0.20	*Heisteria* (Olacaceae)	1
72	*Margaritaria* (Phyllanthaceae)	0.09	0.21	0.08	*Aparisthmium* (Euphorbiaceae)	4	0.16	*Helicostylis* (Moraceae)	1
73	*Apuleia* (Fabaceae)	0.09	0.21	0.71	*Cariniana* (Lecythidaceae)	4	0.16	*Hieronyma* (Phyllanthaceae)	1
74	*Allophylus* (Sapindaceae)	0.08	0.21	0.04	*Cestrum* (Solanaceae)	4	0.16	*Hirtella* (Chrysobalanaceae)	1
75	*Urbanodendron* (Lauraceae)	0.08	0.20	0.91	*Citronella* (Cardiopteridaceae)	4	0.16	*Hortia* (Rutaceae)	1
76	*Miconia* (Melastomataceae)	0.08	0.20	0.28	*Clarisia* (Moraceae)	4	0.16	*Hymenaea* (Fabaceae)	1
77	*Melanoxylon* (Fabaceae)	0.08	0.20	0.08	*Cordia* (Boraginaceae)	4	0.16	*Ilex* (Aquifoliaceae)	1
78	*Lacistema* (Lacistemataceae)	0.08	0.19	0.28	*Coussapoa* (Urticaceae)	4	0.16	*Ixora* (Rubiaceae)	1
79	*Aspidosperma* (Apocynaceae)	0.07	0.18	0.44	*Croton* (Euphorbiaceae)	4	0.16	*Jacaranda* (Bignoniaceae)	1
80	*Tovomita* (Clusiaceae)	0.07	0.17	0.28	*Maclura* (Moraceae)	4	0.16	*Jacaratia* (Caricaceae)	1
81	*Maclura* (Moraceae)	0.07	0.17	0.16	*Sapium* (Euphorbiaceae)	4	0.16	*Kielmeyera* (Calophyllaceae)	1
82	*Maprounea* (Euphorbiaceae)	0.07	0.17	0.08	*Cupania* (Sapindaceae)	3	0.12	*Lacistema* (Lacistemataceae)	1
83	*Lonchocarpus* (Fabaceae)	0.06	0.15	0.04	*Dalbergia* (Fabaceae)	3	0.12	*Lafoensia* (Lythraceae)	1
84	*Ouratea* (Ochnaceae)	0.06	0.15	0.32	*Ficus* (Moraceae)	3	0.12	*Licania* (Chrysobalanaceae)	1
85	*Pera* (Euphorbiaceae)	0.06	0.15	0.08	*Guettarda* (Rubiaceae)	3	0.12	*Lonchocarpus* (Fabaceae)	1
86	*Guettarda* (Rubiaceae)	0.06	0.14	0.12	*Kielmeyera* (Calophyllaceae)	3	0.12	*Luehea* (Malvaceae)	1
87	*Tabernaemontana* (Apocynaceae)	0.06	0.14	0.08	*Mabea* (Euphorbiaceae)	3	0.12	*Mabea* (Euphorbiaceae)	1
88	*Cedrela* (Meliaceae)	0.06	0.14	0.08	*Salacia* (Celastraceae)	3	0.12	*Maclura* (Moraceae)	1
89	*Genipa* (Rubiaceae)	0.05	0.13	0.04	*Andira* (Fabaceae)	2	0.08	*Macrothumia* (Salicaceae)	1
90	*Naucleopsis* (Moraceae)	0.05	0.13	0.04	*Cedrela* (Meliaceae)	2	0.08	*Maprounea* (Euphorbiaceae)	1
91	*Peltophorum* (Fabaceae)	0.05	0.13	0.08	*Erythroxylum* (Erythroxylaceae)	2	0.08	*Margaritaria* (Phyllanthaceae)	1
92	*Mouriri* (Melastomataceae)	0.05	0.13	0.55	*Hieronyma* (Phyllanthaceae)	2	0.08	*Matayba* (Sapindaceae)	1
93	*Luehea* (Malvaceae)	0.05	0.11	0.08	*Hymenaea* (Fabaceae)	2	0.08	*Melanoxylon* (Fabaceae)	1
94	*Tovomitopsis* (Clusiaceae)	0.04	0.10	0.40	*Ilex* (Aquifoliaceae)	2	0.08	*Meliosma* (Sabiaceae)	1
95	*Cariniana* (Lecythidaceae)	0.04	0.10	0.16	*Licania* (Chrysobalanaceae)	2	0.08	*Mollinedia* (Monimiaceae)	1
96	*Xylosma* (Salicaceae)	0.04	0.09	0.08	*Luehea* (Malvaceae)	2	0.08	*Mouriri* (Melastomataceae)	1
97	*Kielmeyera* (Calophyllaceae)	0.03	0.08	0.12	*Maprounea* (Euphorbiaceae)	2	0.08	*Myrciaria* (Myrtaceae)	1
98	*Mabea* (Euphorbiaceae)	0.03	0.08	0.12	*Margaritaria* (Phyllanthaceae)	2	0.08	*Myrsine* (Primulaceae)	1
99	*Aparisthmium* (Euphorbiaceae)	0.03	0.08	0.16	*Melanoxylon* (Fabaceae)	2	0.08	*Naucleopsis* (Moraceae)	1
100	*Heisteria* (Olacaceae)	0.03	0.08	0.28	*Ormosia* (Fabaceae)	2	0.08	*Neomitranthes* (Myrtaceae)	1
101	*Alchornea* (Euphorbiaceae)	0.03	0.08	0.20	*Peltophorum* (Fabaceae)	2	0.08	*Ormosia* (Fabaceae)	1
102	*Garcinia* (Clusiaceae)	0.02	0.06	0.32	*Pera* (Euphorbiaceae)	2	0.08	*Ouratea* (Ochnaceae)	1
103	*Cinnamomum* (Lauraceae)	0.02	0.05	0.04	*Pradosia* (Sapotaceae)	2	0.08	*Peltophorum* (Fabaceae)	1
104	*Vernonanthura* (Asteraceae)	0.02	0.05	0.04	*Randia* (Rubiaceae)	2	0.08	*Pera* (Euphorbiaceae)	1
105	*Cestrum* (Solanaceae)	0.02	0.05	0.16	*Sparattosperma* (Bignoniaceae)	2	0.08	*Phyllostemonodaphne* (Lauraceae)	1
106	*Cryptocarya* (Lauraceae)	0.02	0.05	0.04	*Tabernaemontana* (Apocynaceae)	2	0.08	*Piptadenia* (Fabaceae)	1
107	*Plinia* (Myrtaceae)	0.02	0.05	0.04	*Xylosma* (Salicaceae)	2	0.08	*Pisonia* (Nyctaginaceae)	1
108	*Cupania* (Sapindaceae)	0.02	0.04	0.12	*Zanthoxylum* (Rutaceae)	2	0.08	*Plinia* (Myrtaceae)	1
109	*Myrsine* (Primulaceae)	0.02	0.04	0.20	*Macrothumia* (Salicaceae)	2	0.08	*Pourouma* (Urticaceae)	1
110	*Carpotroche* (Achariaceae)	0.02	0.04	0.24	*Brunfelsia* (Solanaceae)	1	0.04	*Pouteria* (Sapotaceae)	1
111	*Piper* (Piperaceae)	0.01	0.04	0.44	*Cabralea* (Meliaceae)	1	0.04	*Pradosia* (Sapotaceae)	1
112	*Meliosma* (Sabiaceae)	0.01	0.04	0.32	*Campomanesia* (Myrtaceae)	1	0.04	*Prockia* (Salicaceae)	1
113	*Clarisia* (Moraceae)	0.01	0.03	0.16	*Ceiba* (Malvaceae)	1	0.04	*Prunus* (Rosaceae)	1
114	*Prockia* (Salicaceae)	0.01	0.03	0.04	*Celtis* (Cannabaceae)	1	0.04	*Pseudopiptadenia* (Fabaceae)	1
115	*Erythroxylum* (Erythroxylaceae)	0.01	0.03	0.08	*Cinnamomum* (Lauraceae)	1	0.04	*Psidium* (Myrtaceae)	1
116	*Rudgea* (Rubiaceae)	0.01	0.02	0.24	*Colubrina* (Rhamnaceae)	1	0.04	*Qualea* (Vochysiaceae)	1
117	*Citronella* (Cardiopteridaceae)	0.01	0.02	0.16	*Cryptocarya* (Lauraceae)	1	0.04	*Randia* (Rubiaceae)	1
118	*Hortia* (Rutaceae)	0.01	0.02	0.04	*Cybianthus* (Primulaceae)	1	0.04	*Rudgea* (Rubiaceae)	1
119	*Mollinedia* (Monimiaceae)	0.01	0.02	0.20	*Dendropanax* (Araliaceae)	1	0.04	*Salacia* (Celastraceae)	1
120	*Cabralea* (Meliaceae)	0.01	0.02	0.04	*Genipa* (Rubiaceae)	1	0.04	*Sapium* (Euphorbiaceae)	1
121	*Sparattosperma* (Bignoniaceae)	0.01	0.02	0.08	*Handroanthus* (Bignoniaceae)	1	0.04	*Schefflera* (Araliaceae)	1
122	*Ilex* (Aquifoliaceae)	0.01	0.02	0.08	*Hortia* (Rutaceae)	1	0.04	*Sloanea* (Elaeocarpaceae)	1
123	*Sloanea* (Elaeocarpaceae)	0.01	0.02	0.04	*Jacaratia* (Caricaceae)	1	0.04	*Sparattosperma* (Bignoniaceae)	1
124	*Prunus* (Rosaceae)	0.00	0.01	0.04	*Lonchocarpus* (Fabaceae)	1	0.04	*Sterculia* (Malvaceae)	1
125	*Dendropanax* (Araliaceae)	0.00	0.01	0.04	*Allophylus* (Sapindaceae)	1	0.04	*Stylogyne* (Primulaceae)	1
126	*Campomanesia* (Myrtaceae)	0.00	0.01	0.04	*Naucleopsis* (Moraceae)	1	0.04	*Tabernaemontana* (Apocynaceae)	1
127	*Jacaratia* (Caricaceae)	0.00	0.01	0.04	*Plinia* (Myrtaceae)	1	0.04	*Terminalia* (Combretaceae)	1
128	*Stylogyne* (Primulaceae)	0.00	0.01	0.04	*Prockia* (Salicaceae)	1	0.04	*Tetrastylidium* (Olacaceae)	1
129	*Terminalia* (Combretaceae)	0.00	0.01	0.04	*Prunus* (Rosaceae)	1	0.04	*Tovomita* (Clusiaceae)	1
130	*Brunfelsia* (Solanaceae)	0.00	0.01	0.04	*Psidium* (Myrtaceae)	1	0.04	*Tovomitopsis* (Clusiaceae)	1
131	*Randia* (Rubiaceae)	0.00	0.01	0.08	*Qualea* (Vochysiaceae)	1	0.04	*Trattinnickia* (Burseraceae)	1
132	*Psidium* (Myrtaceae)	0.00	0.01	0.04	*Sloanea* (Elaeocarpaceae)	1	0.04	*Urbanodendron* (Lauraceae)	1
133	*Celtis* (Cannabaceae)	0.00	0.00	0.04	*Stylogyne* (Primulaceae)	1	0.04	*Vantanea* (Humiriaceae)	1
134	*Vantanea* (Humiriaceae)	0.00	0.00	0.04	*Terminalia* (Combretaceae)	1	0.04	*Vernonanthura* (Asteraceae)	1
135	*Handroanthus* (Bignoniaceae)	0.00	0.00	0.04	*Vantanea* (Humiriaceae)	1	0.04	*Xylosma* (Salicaceae)	1
136	*Cybianthus* (Primulaceae)	0.00	0.00	0.04	*Vernonanthura* (Asteraceae)	1	0.04	*Zanthoxylum* (Rutaceae)	1
137	Unidentified (14 morphospecies)	0.37	0.93	0.91	Unidentified (14 morphospecies)	23	0.91	Unidentified	14

**Table 5.** FSN tree species ≥ 3.2 cm dbh ranked by number of trees (N) and basal area (BA).

**Rank**	**Species**	**BA**	**% BA**	**N**	**Species**	**N**	**%**
1	*Ficus gomelleira* Kunth & C.D. Bouché (Moraceae)	3.41	8.48	1	*Siparuna guianensis* Aubl. (Siparunaceae)	254	10.04
2	*Pseudopiptadenia contorta* (DC.) G.P. Lewis & M.P. Lima (Fabaceae)	3.08	7.67	45	*Protium warmingiana* March,L. (Burseraceae)	121	4.78
3	*Virola gardneri* (A. DC.) Warb. (Myristicaceae)	2.21	5.51	77	*Sorocea bonplandii* (Baill.) W.C. Burger, Lanj. & Wess. Boer (Moraceae)	107	4.23
4	*Guatteria australis* A. St.-Hil. (Annonaceae)	1.91	4.76	67	*Myrciaria floribunda* (H. West ex Willd.) O. Berg (Myrtaceae)	101	3.99
5	*Sterculia curiosa* (Vell.) Taroda (Malvaceae)	1.84	4.58	12	*Euterpe edulis* Mart. (Arecaceae)	90	3.56
6	*Ceiba speciosa* (A. St.-Hil.) Ravenna (Malvaceae)	1.37	3.41	1	*Virola gardneri* (A. DC.) Warb. (Myristicaceae)	77	3.04
7	*Protium warmingiana* March,L. (Burseraceae)	1.29	3.22	121	*Helicostylis tomentosa* (Poepp. & Endl.) Rusby (Moraceae)	73	2.89
8	*Sorocea bonplandii* (Baill.) W.C. Burger, Lanj. & Wess. Boer (Moraceae)	1.26	3.13	107	*Guatteria australis* A. St.-Hil. (Annonaceae)	67	2.65
9	*Astronium graveolens* Jacq. (Anacardiaceae)	0.97	2.43	13	*Bathysa nicholsonii* K. Schum. (Rubiaceae)	60	2.37
10	*Ocotea silvestris* Vattimo (Lauraceae)	0.82	2.05	24	*Marlierea excoriata* Mart. (Myrtaceae)	59	2.33
11	*Casearia ulmifolia* Vahl ex Vent. (Salicaceae)	0.73	1.81	30	*Phyllostemonodaphne geminiflora* (Mez) Kosterm. (Lauraceae)	59	2.33
12	*Pourouma guianensis* Aubl. (Urticaceae)	0.66	1.64	31	*Brosimum guianense* (Aubl.) Huber (Moraceae)	49	1.94
13	*Helicostylis tomentosa* (Poepp. & Endl.) Rusby (Moraceae)	0.64	1.60	73	*Eugenia lambertiana* DC. (Myrtaceae)	47	1.86
14	*Euterpe edulis* Mart. (Arecaceae)	0.64	1.59	90	*Pseudopiptadenia contorta* (DC.) G.P. Lewis & M.P. Lima (Fabaceae)	45	1.78
15	*Bathysa nicholsonii* K. Schum. (Rubiaceae)	0.60	1.48	60	*Trichilia glabra* L. (Meliaceae)	35	1.38
16	*Brosimum guianense* (Aubl.) Huber (Moraceae)	0.59	1.48	49	*Trichilia catigua* A. Juss. (Meliaceae)	34	1.34
17	*Siparuna guianensis* Aubl. (Siparunaceae)	0.58	1.44	254	*Alseis floribunda* Schott (Rubiaceae)	31	1.23
18	*Virola bicuhyba* (Schott ex Spreng.) Warb. (Myristicaceae)	0.56	1.40	19	*Pourouma guianensis* Aubl. (Urticaceae)	31	1.23
19	*Guarea macrophylla* Vahl (Meliaceae)	0.56	1.38	15	*Casearia ulmifolia* Vahl ex Vent. (Salicaceae)	30	1.19
20	*Ocotea odorifera* Rohwer (Lauraceae)	0.54	1.36	27	*Chrysophyllum lucentifolium* Cronquist (Sapotaceae)	28	1.11
21	*Trattinnickia* sp. (Burseraceae)	0.51	1.28	6	*Ocotea odorifera* Rohwer (Lauraceae)	27	1.07
22	*Eriotheca candolleana* (K. Schum.) A. Robyns (Malvaceae)	0.50	1.25	13	*Neomitranthes* sp. (Myrtaceae)	26	1.03
23	*Trichilia catigua* A. Juss. (Meliaceae)	0.48	1.21	34	*Maytenus robusta* Reissek (Celastraceae)	25	0.99
24	*Inga cylindrica* (Vell.) Mart. (Fabaceae)	0.45	1.11	7	*Chrysophyllum gonocarpum* (Mart. & Eichler ex Miq.) Engl. (Sapotaceae)	24	0.95
25	*Piptadenia gonoacantha* (Mart.) J.F. Macbr. (Fabaceae)	0.38	0.94	6	*Inga capitata* Desv. (Fabaceae)	24	0.95
26	*Coussapoa floccosa* Akkermans & C.C. Berg (Urticaceae)	0.35	0.88	1	*Myrcia laxiflora* Cambess. (Myrtaceae)	24	0.95
27	*Cecropia hololeuca* Miq. (Urticaceae)	0.35	0.86	9	*Ocotea dispersa* (Nees & Mart.) Mez (Lauraceae)	24	0.95
28	*Matayba elaeagnoides* Radlk. (Sapindaceae)	0.35	0.86	12	*Ocotea silvestris* Vattimo (Lauraceae)	24	0.95
29	*Sorocea guilleminiana* Gaudich. (Moraceae)	0.34	0.84	19	*Pouteria caimito* (Ruiz & Pav.) Radlk. (Sapotaceae)	24	0.95
30	*Myrciaria floribunda* (H. West ex Willd.) O. Berg (Myrtaceae)	0.33	0.83	101	*Urbanodendron verrucosum* (Nees) Mez (Lauraceae)	23	0.91
31	*Annona cacans* Warm. (Annonaceae)	0.31	0.77	4	*Aniba firmula* (Nees & Mart.) Mez (Lauraceae)	22	0.87
32	*Phyllostemonodaphne geminiflora* (Mez) Kosterm. (Lauraceae)	0.30	0.74	59	*Marlierea teuscheriana* (O. Berg) D. Legrand (Myrtaceae)	21	0.83
33	*Annona neolaurifolia* H. Rainer (Annonaceae)	0.29	0.73	6	*Myrcia splendens* (Sw.) DC. (Myrtaceae)	21	0.83
34	*Eugenia lambertiana* DC. (Myrtaceae)	0.29	0.73	47	*Calyptranthes brasiliensis* Spreng. (Myrtaceae)	20	0.79
35	*Chrysophyllum lucentifolium* Cronquist (Sapotaceae)	0.27	0.68	28	*Tetrastylidium grandifolium* (Baill.) Sleumer (Olacaceae)	20	0.79
36	*Machaerium nyctitans* (Vell.) Benth. (Fabaceae)	0.25	0.63	8	*Ocotea* sp. (Lauraceae)	19	0.75
37	*Hirtella hebeclada* Moric. ex DC. (Chrysobalanaceae)	0.24	0.59	13	*Sorocea guilleminiana* Gaudich. (Moraceae)	19	0.75
38	*Tetrastylidium grandifolium* (Baill.) Sleumer (Olacaceae)	0.23	0.57	20	*Virola bicuhyba* (Schott ex Spreng.) Warb. (Myristicaceae)	19	0.75
39	*Cordia sellowiana* Cham. (Boraginaceae)	0.21	0.53	4	*Amaioua guianensis* Aubl. (Rubiaceae)	18	0.71
40	*Jacaranda macrantha* Cham. (Bignoniaceae)	0.21	0.52	10	*Apuleia leiocarpa* (Vogel) J.F. Macbr. (Fabaceae)	18	0.71
41	*Maytenus robusta* Reissek (Celastraceae)	0.20	0.50	25	*Copaifera langsdorffii* Desf. (Fabaceae)	15	0.59
42	*Macrothumia kuhlmannii* (Sleumer) Alford (Salicaceae)	0.19	0.48	2	*Guarea macrophylla* Vahl (Meliaceae)	15	0.59
43	*Amaioua guianensis* Aubl. (Rubiaceae)	0.18	0.45	18	*Swartzia myrtifolia* Sm. (Fabaceae)	15	0.59
44	*Ormosia arborea* (Vell.) Harms (Fabaceae)	0.18	0.44	2	*Mouriri glazioviana* Cogn. (Melastomataceae)	14	0.55
45	*Chrysophyllum gonocarpum* (Mart. & Eichler ex Miq.) Engl. (Sapotaceae)	0.18	0.44	24	*Astronium graveolens* Jacq. (Anacardiaceae)	13	0.51
46	*Ixora gardneriana* Benth. (Rubiaceae)	0.18	0.44	12	*Eriotheca candolleana* (K. Schum.) A. Robyns (Malvaceae)	13	0.51
47	*Copaifera langsdorffii* Desf. (Fabaceae)	0.17	0.41	15	*Hirtella hebeclada* Moric. ex DC. (Chrysobalanaceae)	13	0.51
48	*Neomitranthes* sp. (Myrtaceae)	0.17	0.41	26	*Myrcia pallida* (O. Berg) N. Silveira (Myrtaceae)	13	0.51
49	*Ocotea dispersa* (Nees & Mart.) Mez (Lauraceae)	0.16	0.40	24	*Ixora gardneriana* Benth. (Rubiaceae)	12	0.47
50	*Trichilia glabra* L. (Meliaceae)	0.16	0.40	35	*Matayba elaeagnoides* Radlk. (Sapindaceae)	12	0.47
51	*Hymenaea* sp. (Fabaceae)	0.16	0.39	2	*Sterculia curiosa* (Vell.) Taroda (Malvaceae)	12	0.47
52	*Andira fraxinifolia* Benth. (Fabaceae)	0.16	0.39	2	*Guapira opposita* (Vell.) Reitz (Nyctaginaceae)	11	0.43
53	Fabaceae sp.	0.15	0.38	1	*Guarea pendula* R.da Silva Ramalho, A.L. Pinheiro & T.D. Penn. (Meliaceae)	11	0.43
54	*Alseis floribunda* Schott (Rubiaceae)	0.15	0.37	31	*Myrcia pubipetala* Miq. (Myrtaceae)	11	0.43
55	*Casearia gossypiosperma* Briq. (Salicaceae)	0.15	0.37	6	*Eugenia florida* DC. (Myrtaceae)	10	0.40
56	*Tapirira guianensis* Aubl. (Anacardiaceae)	0.14	0.35	9	*Jacaranda macrantha* Cham. (Bignoniaceae)	10	0.40
57	*Sapium glandulosum* (L.) Morong (Euphorbiaceae)	0.14	0.35	4	*Pisonia ambigua* Heimerl (Nyctaginaceae)	10	0.40
58	*Marlierea excoriata* Mart. (Myrtaceae)	0.14	0.34	59	*Psychotria carthagenensis* Jacq. (Rubiaceae)	10	0.40
59	*Inga capitata* Desv. (Fabaceae)	0.14	0.34	24	*Tovomitopsis saldanhae* Engl. (Clusiaceae)	10	0.40
60	*Casearia arborea* (Rich.) Urb. (Salicaceae)	0.14	0.34	9	*Casearia arborea* (Rich.) Urb. (Salicaceae)	9	0.36
61	*Lafoensia glyptocarpa* Koehne (Lythraceae)	0.14	0.34	8	*Cecropia hololeuca* Miq. (Urticaceae)	9	0.36
62	*Astrocaryum aculeatissimum* (Schott) Burret (Arecaceae)	0.13	0.33	5	*Piper arboreum* Aubl. (Piperaceae)	9	0.36
63	*Coussapoa microcarpa* (Schott) Rizzini (Urticaceae)	0.13	0.33	3	*Tapirira guianensis* Aubl. (Anacardiaceae)	9	0.36
64	*Aniba firmula* (Nees & Mart.) Mez (Lauraceae)	0.12	0.31	22	*Garcinia gardneriana* (Planch. & Triana) Zappi (Clusiaceae)	8	0.32
65	*Zanthoxylum rhoifolium* Lam. (Rutaceae)	0.12	0.30	2	*Lafoensia glyptocarpa* Koehne (Lythraceae)	8	0.32
66	*Calyptranthes brasiliensis* Spreng. (Myrtaceae)	0.12	0.30	20	*Machaerium nyctitans* (Vell.) Benth. (Fabaceae)	8	0.32
67	*Pisonia ambigua* Heimerl (Nyctaginaceae)	0.12	0.29	10	*Meliosma itatiaiae* Urb. (Sabiaceae)	8	0.32
68	*Dalbergia nigra* (Vell.) Allemão ex Benth. (Fabaceae)	0.11	0.28	3	*Ocotea corymbosa* (Meisn.) Mez (Lauraceae)	8	0.32
69	Salicaceae sp.	0.11	0.28	5	*Ouratea polygyna* Engl. (Ochnaceae)	8	0.32
70	*Myrcia splendens* (Sw.) DC. (Myrtaceae)	0.11	0.28	21	*Protium heptaphyllum* (Aubl.) Marchand (Burseraceae)	8	0.32
71	*Croton floribundus* Spreng. (Euphorbiaceae)	0.11	0.28	4	*Siparuna reginae* (Tul.) A. DC. (Siparunaceae)	8	0.32
72	*Salacia elliptica* (Mart. ex Schult.) G. Don (Celastraceae)	0.11	0.27	3	*Bathysa cuspidata* (A. St.-Hil.) Hook. f. ex K. Schum. (Rubiaceae)	7	0.28
73	*Ocotea* sp. (Lauraceae)	0.10	0.26	19	*Heisteria silvianii* Schwacke (Olacaceae)	7	0.28
74	*Hieronyma alchorneoides* Allemão (Phyllanthaceae)	0.10	0.25	2	*Inga cylindrica* (Vell.) Mart. (Fabaceae)	7	0.28
75	*Pouteria caimito* (Ruiz & Pav.) Radlk. (Sapotaceae)	0.10	0.24	24	*Lacistema pubescens* Mart. (Lacistemataceae)	7	0.28
76	*Schefflera morototoni* (Aubl.) Maguire, Steyerm. & Frodin (Araliaceae)	0.10	0.24	6	*Nectandra lanceolata* Nees & Mart. (Lauraceae)	7	0.28
77	*Licania belemii* Prance (Chrysobalanaceae)	0.09	0.23	2	*Tapirira obtusa* (Benth.) J.D. Mitch. (Anacardiaceae)	7	0.28
78	*Pradosia lactescens* (Vell.) Radlk. (Sapotaceae)	0.09	0.23	2	*Tovomita glazioviana* Engl. (Clusiaceae)	7	0.28
79	*Marlierea teuscheriana* (O. Berg) D. Legrand (Myrtaceae)	0.09	0.23	21	*Annona neolaurifolia* H. Rainer (Annonaceae)	6	0.24
80	*Colubrina glandulosa* Perkins (Rhamnaceae)	0.09	0.22	1	*Carpotroche brasiliensis* (Raddi) Endl. (Achariaceae)	6	0.24
81	*Qualea multiflora* Mart. (Vochysiaceae)	0.09	0.22	1	*Casearia gossypiosperma* Briq. (Salicaceae)	6	0.24
82	*Margaritaria nobilis* L. f. (Phyllanthaceae)	0.09	0.21	2	*Piptadenia gonoacantha* (Mart.) J.F. Macbr. (Fabaceae)	6	0.24
83	*Apuleia leiocarpa* (Vogel) J.F. Macbr. (Fabaceae)	0.09	0.21	18	*Rudgea myrsinifolia* Benth. (Rubiaceae)	6	0.24
84	*Bathysa cuspidata* (A. St.-Hil.) Hook. f. ex K. Schum. (Rubiaceae)	0.09	0.21	7	*Schefflera morototoni* (Aubl.) Maguire, Steyerm. & Frodin (Araliaceae)	6	0.24
85	*Allophylus edulis* (A. St.-Hil., A. Juss. & Cambess.) Hieron. ex Niederl. (Sapindaceae)	0.08	0.21	1	*Trattinnickia* sp. (Burseraceae)	6	0.24
86	*Nectandra lanceolata* Nees & Mart. (Lauraceae)	0.08	0.21	7	*Aspidosperma polyneuron* Müll. Arg. (Apocynaceae)	5	0.20
87	*Protium heptaphyllum* (Aubl.) Marchand (Burseraceae)	0.08	0.21	8	*Astrocaryum aculeatissimum* (Schott) Burret (Arecaceae)	5	0.20
88	*Urbanodendron verrucosum* (Nees) Mez (Lauraceae)	0.08	0.20	23	*Guatteria* sp.1 (Annonaceae)	5	0.20
89	*Astronium fraxinifolium* Schott ex Spreng. (Anacardiaceae)	0.08	0.20	4	*Mollinedia schottiana* (Spreng.) Perkins (Monimiaceae)	5	0.20
90	*Psychotria carthagenensis* Jacq. (Rubiaceae)	0.08	0.20	10	*Myrsine umbellata* Mart. (Primulaceae)	5	0.20
91	*Melanoxylon brauna* Schott (Fabaceae)	0.08	0.20	2	*Psychotria nuda* (Cham. & Schltdl.) Wawra (Rubiaceae)	5	0.20
92	*Lacistema pubescens* Mart. (Lacistemataceae)	0.08	0.19	7	Salicaceae sp.	5	0.20
93	*Tapirira obtusa* (Benth.) J.D. Mitch. (Anacardiaceae)	0.08	0.19	7	*Trichilia lepidota* Mart. (Meliaceae)	5	0.20
94	*Xylopia brasiliensis* Spreng. (Annonaceae)	0.07	0.19	3	*Annona cacans* Warm. (Annonaceae)	4	0.16
95	*Ocotea corymbosa* (Meisn.) Mez (Lauraceae)	0.07	0.18	8	*Aparisthmium cordatum* (A. Juss.) Baill. (Euphorbiaceae)	4	0.16
96	*Guapira opposita* (Vell.) Reitz (Nyctaginaceae)	0.07	0.17	11	*Astronium fraxinifolium* Schott ex Spreng. (Anacardiaceae)	4	0.16
97	*Myrcia pallida* (O. Berg) N. Silveira (Myrtaceae)	0.07	0.17	13	*Citronella megaphylla* (Miers) R.A. Howard (Cardiopteridaceae)	4	0.16
98	*Tovomita glazioviana* Engl. (Clusiaceae)	0.07	0.17	7	*Clarisia ilicifolia* (Spreng.) Lanj. & Rossberg (Moraceae)	4	0.16
99	*Myrcia pubipetala* Miq. (Myrtaceae)	0.07	0.17	11	*Cordia sellowiana* Cham. (Boraginaceae)	4	0.16
100	*Maclura tinctoria* (L.) D. Don ex Steud. (Moraceae)	0.07	0.17	4	*Croton floribundus* Spreng. (Euphorbiaceae)	4	0.16
101	*Maprounea guianensis* Aubl. (Euphorbiaceae)	0.07	0.17	2	*Eugenia leptoclada* O. Berg (Myrtaceae)	4	0.16
102	*Swartzia myrtifolia* Sm. (Fabaceae)	0.06	0.16	15	*Guapira hirsuta* (Choisy) Lundell (Nyctaginaceae)	4	0.16
103	*Lonchocarpus cultratus* (Vell.) A.M.G. Azevedo & H.C. Lima (Fabaceae)	0.06	0.15	1	*Maclura tinctoria* (L.) D. Don ex Steud. (Moraceae)	4	0.16
104	*Ouratea polygyna* Engl. (Ochnaceae)	0.06	0.15	8	*Sapium glandulosum* (L.) Morong (Euphorbiaceae)	4	0.16
105	*Pera glabrata* (Schott) Poepp. ex Baill. (Euphorbiaceae)	0.06	0.15	2	*Alchornea glandulosa* Poepp. (Euphorbiaceae)	3	0.12
106	*Guettarda viburnoides* Cham. & Schltdl. (Rubiaceae)	0.06	0.14	3	*Aspidosperma olivaceum* Müll. Arg. (Apocynaceae)	3	0.12
107	*Tabernaemontana fuchsiaefolia* A. DC. (Apocynaceae)	0.06	0.14	2	*Aspidosperma subincanum* Mart. ex A. DC. (Apocynaceae)	3	0.12
108	*Trichilia lepidota* Mart. (Meliaceae)	0.06	0.14	5	*Cariniana estrellensis* (Raddi) Kuntze (Lecythidaceae)	3	0.12
109	*Aspidosperma polyneuron* Müll. Arg. (Apocynaceae)	0.06	0.14	5	*Coussapoa microcarpa* (Schott) Rizzini (Urticaceae)	3	0.12
110	*Cedrela fissilis* Vell. (Meliaceae)	0.06	0.14	2	*Cupania vernalis* Cambess. (Sapindaceae)	3	0.12
111	*Genipa americana* L. (Rubiaceae)	0.05	0.13	1	*Dalbergia nigra* (Vell.) Allemão ex Benth. (Fabaceae)	3	0.12
112	*Naucleopsis oblongifolia* (Kuhlm.) Carauta (Moraceae)	0.05	0.13	1	*Eugenia dodonaeifolia* Cambess. (Myrtaceae)	3	0.12
113	*Peltophorum dubium* (Spreng.) Taub. (Fabaceae)	0.05	0.13	2	*Guettarda viburnoides* Cham. & Schltdl. (Rubiaceae)	3	0.12
114	*Mouriri glazioviana* Cogn. (Melastomataceae)	0.05	0.13	14	*Kielmeyera albopunctata* Saddi (Calophyllaceae)	3	0.12
115	Unidentified sp.4	0.05	0.12	1	*Mabea fistulifera* Mart. (Euphorbiaceae)	3	0.12
116	*Marlierea suaveolens* Cambess. (Myrtaceae)	0.05	0.11	1	*Maytenus salicifolia* Reissek (Celastraceae)	3	0.12
117	*Luehea grandiflora* Mart. (Malvaceae)	0.05	0.11	2	*Miconia budlejoides* Triana (Melastomataceae)	3	0.12
118	*Miconia cinnamomifolia* (DC.) Naudin (Melastomataceae)	0.04	0.11	1	*Nectandra oppositifolia* Nees & Mart. (Lauraceae)	3	0.12
119	*Myrcia laxiflora* Cambess. (Myrtaceae)	0.04	0.11	24	Nyctaginaceae sp.	3	0.12
120	*Xylopia sericea* A. St.-Hil. (Annonaceae)	0.04	0.11	3	*Salacia elliptica* (Mart. ex Schult.) G. Don (Celastraceae)	3	0.12
121	*Guatteria* sp.1 (Annonaceae)	0.04	0.10	5	*Xylopia brasiliensis* Spreng. (Annonaceae)	3	0.12
122	*Tovomitopsis saldanhae* Engl. (Clusiaceae)	0.04	0.10	10	*Xylopia sericea* A. St.-Hil. (Annonaceae)	3	0.12
123	*Nectandra oppositifolia* Nees & Mart. (Lauraceae)	0.04	0.10	3	*Alchornea triplinervia* (Spreng.) Müll. Arg. (Euphorbiaceae)	2	0.08
124	*Xylosma prockia* (Turcz.) Turcz. (Salicaceae)	0.04	0.09	2	*Andira fraxinifolia* Benth. (Fabaceae)	2	0.08
125	*Trichilia pallida* Sw. (Meliaceae)	0.04	0.09	2	*Casearia sylvestris* Sw. (Salicaceae)	2	0.08
126	*Kielmeyera albopunctata* Saddi (Calophyllaceae)	0.03	0.08	3	*Cedrela fissilis* Vell. (Meliaceae)	2	0.08
127	*Mabea fistulifera* Mart. (Euphorbiaceae)	0.03	0.08	3	*Cestrum mariquitense* Kunth (Solanaceae)	2	0.08
128	*Aparisthmium cordatum* (A. Juss.) Baill. (Euphorbiaceae)	0.03	0.08	4	*Cestrum* sp. (Solanaceae)	2	0.08
129	*Inga* sp. (Fabaceae)	0.03	0.08	1	*Chrysophyllum marginatum* (Hook. & Arn.) Radlk. (Sapotaceae)	2	0.08
130	*Heisteria silvianii* Schwacke (Olacaceae)	0.03	0.08	7	*Chrysophyllum* sp. (Sapotaceae)	2	0.08
131	*Cariniana legalis* (Mart.) Kuntze (Lecythidaceae)	0.03	0.07	1	Euphorbiaceae sp.2	2	0.08
132	*Psychotria nuda* (Cham. & Schltdl.) Wawra (Rubiaceae)	0.03	0.07	5	*Hieronyma alchorneoides* Allemão (Phyllanthaceae)	2	0.08
133	*Swartzia acutifolia* Vogel (Fabaceae)	0.03	0.07	1	*Hymenaea* sp. (Fabaceae)	2	0.08
134	*Miconia budlejoides* Triana (Melastomataceae)	0.03	0.07	3	Ilex cerasifolia var. glaziovii Loes. (Aquifoliaceae)	2	0.08
135	*Garcinia gardneriana* (Planch. & Triana) Zappi (Clusiaceae)	0.02	0.06	8	*Inga vera* Willd. (Fabaceae)	2	0.08
136	*Ocotea pulchella* (Nees & Mart.) Mez (Lauraceae)	0.02	0.06	1	*Licania belemii* Prance (Chrysobalanaceae)	2	0.08
137	*Siparuna reginae* (Tul.) A. DC. (Siparunaceae)	0.02	0.06	8	*Luehea grandiflora* Mart. (Malvaceae)	2	0.08
138	*Chrysophyllum marginatum* (Hook. & Arn.) Radlk. (Sapotaceae)	0.02	0.05	2	*Macrothumia kuhlmannii* (Sleumer) Alford (Salicaceae)	2	0.08
139	*Cinnamomum glaziovii* (Mez) Kosterm. (Lauraceae)	0.02	0.05	1	*Maprounea guianensis* Aubl. (Euphorbiaceae)	2	0.08
140	*Alchornea triplinervia* (Spreng.) Müll. Arg. (Euphorbiaceae)	0.02	0.05	2	*Margaritaria nobilis* L. f. (Phyllanthaceae)	2	0.08
141	*Vernonia diffusa* Less. (Asteraceae)	0.02	0.05	1	*Melanoxylon brauna* Schott (Fabaceae)	2	0.08
142	*Cryptocarya moschata* Nees & Mart. (Lauraceae)	0.02	0.05	1	*Myrcia anceps* O. Berg (Myrtaceae)	2	0.08
143	*Guapira hirsuta* (Choisy) Lundell (Nyctaginaceae)	0.02	0.05	4	Myrtaceae sp.	2	0.08
144	*Inga vera* Willd. (Fabaceae)	0.02	0.05	2	*Ormosia arborea* (Vell.) Harms (Fabaceae)	2	0.08
145	*Guatteria villosissima* A. St.-Hil. (Annonaceae)	0.02	0.05	1	*Peltophorum dubium* (Spreng.) Taub. (Fabaceae)	2	0.08
146	*Plinia grandifolia* (Mattos) Sobral (Myrtaceae)	0.02	0.05	1	*Pera glabrata* (Schott) Poepp. ex Baill. (Euphorbiaceae)	2	0.08
147	*Cupania vernalis* Cambess. (Sapindaceae)	0.02	0.04	3	*Piper cernuum* Vell. (Piperaceae)	2	0.08
148	*Myrsine umbellata* Mart. (Primulaceae)	0.02	0.04	5	*Pradosia lactescens* (Vell.) Radlk. (Sapotaceae)	2	0.08
149	*Carpotroche brasiliensis* (Raddi) Endl. (Achariaceae)	0.02	0.04	6	*Psychotria* sp. (Rubiaceae)	2	0.08
150	Nyctaginaceae sp.	0.02	0.04	3	*Randia ferox* (Cham. & Schltdl.) DC. (Rubiaceae)	2	0.08
151	*Ficus luschnathiana* (Miq.) Miq. (Moraceae)	0.02	0.04	1	Rubiaceae sp.	2	0.08
152	*Guarea pendula* R.da Silva Ramalho, A.L. Pinheiro & T.D. Penn. (Meliaceae)	0.02	0.04	11	*Sparattosperma leucanthum* (Vell.) K. Schum. (Bignoniaceae)	2	0.08
153	*Casearia sylvestris* Sw. (Salicaceae)	0.02	0.04	2	*Tabernaemontana fuchsiaefolia* A. DC. (Apocynaceae)	2	0.08
154	*Cestrum mariquitense* Kunth (Solanaceae)	0.02	0.04	2	*Trichilia pallida* Sw. (Meliaceae)	2	0.08
155	*Meliosma itatiaiae* Urb. (Sabiaceae)	0.01	0.04	8	*Xylosma prockia* (Turcz.) Turcz. (Salicaceae)	2	0.08
156	Myrtaceae sp.	0.01	0.04	2	*Zanthoxylum rhoifolium* Lam. (Rutaceae)	2	0.08
157	*Eugenia florida* DC. (Myrtaceae)	0.01	0.03	10	*Allophylus edulis* (A. St.-Hil., A. Juss. & Cambess.) Hieron. ex Niederl. (Sapindaceae)	1	0.04
158	*Clarisia ilicifolia* (Spreng.) Lanj. & Rossberg (Moraceae)	0.01	0.03	4	*Brunfelsia uniflora* (Pohl) D. Don (Solanaceae)	1	0.04
159	*Guarea guidonia* (L.) Sleumer (Meliaceae)	0.01	0.03	1	*Cabralea cangerana* Saldanha (Meliaceae)	1	0.04
160	*Prockia crucis* P. Browne ex L. (Salicaceae)	0.01	0.03	1	*Campomanesia xanthocarpa* Mart. ex O. Berg (Myrtaceae)	1	0.04
161	*Piper arboreum* Aubl. (Piperaceae)	0.01	0.03	9	*Cariniana legalis* (Mart.) Kuntze (Lecythidaceae)	1	0.04
162	*Eugenia leptoclada* O. Berg (Myrtaceae)	0.01	0.03	4	*Casearia decandra* Jacq. (Salicaceae)	1	0.04
163	*Maytenus salicifolia* Reissek (Celastraceae)	0.01	0.03	3	*Ceiba speciosa* (A. St.-Hil.) Ravenna (Malvaceae)	1	0.04
164	*Aspidosperma subincanum* Mart. ex A. DC. (Apocynaceae)	0.01	0.02	3	*Celtis iguanaea* (Jacq.) Sarg. (Cannabaceae)	1	0.04
165	*Eugenia dodonaeifolia* Cambess. (Myrtaceae)	0.01	0.02	3	*Cinnamomum glaziovii* (Mez) Kosterm. (Lauraceae)	1	0.04
166	*Alchornea glandulosa* Poepp. (Euphorbiaceae)	0.01	0.02	3	*Colubrina glandulosa* Perkins (Rhamnaceae)	1	0.04
167	*Cariniana estrellensis* (Raddi) Kuntze (Lecythidaceae)	0.01	0.02	3	*Coussapoa floccosa* Akkermans & C.C. Berg (Urticaceae)	1	0.04
168	*Rudgea myrsinifolia* Benth. (Rubiaceae)	0.01	0.02	6	*Cryptocarya moschata* Nees & Mart. (Lauraceae)	1	0.04
169	*Citronella megaphylla* (Miers) R.A. Howard (Cardiopteridaceae)	0.01	0.02	4	*Cybianthus fuscus* Mart. (Primulaceae)	1	0.04
170	*Erythroxylum daphnites* Mart. (Erythroxylaceae)	0.01	0.02	1	*Dendropanax cuneatus* (DC.) Decne. & Planch. (Araliaceae)	1	0.04
171	*Hortia brasiliana* Vand. ex DC. (Rutaceae)	0.01	0.02	1	*Erythroxylum daphnites* Mart. (Erythroxylaceae)	1	0.04
172	*Ficus enormis* (Mart. ex Miq.) Mart. (Moraceae)	0.01	0.02	1	*Erythroxylum pelleterianum* A. St.-Hil. (Erythroxylaceae)	1	0.04
173	*Mollinedia schottiana* (Spreng.) Perkins (Monimiaceae)	0.01	0.02	5	Euphorbiaceae sp.1	1	0.04
174	*Cabralea cangerana* Saldanha (Meliaceae)	0.01	0.02	1	Euphorbiaceae sp.3	1	0.04
175	Unidentified sp.3	0.01	0.02	1	Fabaceae sp.	1	0.04
176	*Sparattosperma leucanthum* (Vell.) K. Schum. (Bignoniaceae)	0.01	0.02	2	*Ficus enormis* (Mart. ex Miq.) Mart. (Moraceae)	1	0.04
177	Ilex cerasifolia var. glaziovii Loes. (Aquifoliaceae)	0.01	0.02	2	*Ficus gomelleira* Kunth & C.D. Bouché (Moraceae)	1	0.04
178	*Sloanea hirsuta* (Schott) Planch. ex Benth. (Elaeocarpaceae)	0.01	0.02	1	*Ficus luschnathiana* (Miq.) Miq. (Moraceae)	1	0.04
179	*Aspidosperma olivaceum* Müll. Arg. (Apocynaceae)	0.01	0.02	3	*Genipa americana* L. (Rubiaceae)	1	0.04
180	*Myrcia anceps* O. Berg (Myrtaceae)	0.01	0.01	2	*Guarea fistulosa* W. Palacios (Meliaceae)	1	0.04
181	*Prunus sellowii* Koehne (Rosaceae)	0.00	0.01	1	*Guarea guidonia* (L.) Sleumer (Meliaceae)	1	0.04
182	*Dendropanax cuneatus* (DC.) Decne. & Planch. (Araliaceae)	0.00	0.01	1	*Guatteria* sp.2 (Annonaceae)	1	0.04
183	*Cestrum* sp. (Solanaceae)	0.00	0.01	2	*Guatteria villosissima* A. St.-Hil. (Annonaceae)	1	0.04
184	*Chrysophyllum* sp. (Sapotaceae)	0.00	0.01	2	*Handroanthus chrysotrichus* (Mart. ex A. DC.) Mattos (Bignoniaceae)	1	0.04
185	*Guarea fistulosa* W. Palacios (Meliaceae)	0.00	0.01	1	*Hortia brasiliana* Vand. ex DC. (Rutaceae)	1	0.04
186	*Miconia brunnea* Mart. ex DC. (Melastomataceae)	0.00	0.01	1	*Inga* sp. (Fabaceae)	1	0.04
187	*Campomanesia xanthocarpa* Mart. ex O. Berg (Myrtaceae)	0.00	0.01	1	*Jacaratia heptaphylla* (Vell.) A. DC. (Caricaceae)	1	0.04
188	*Machaerium caratinganum* Kuhlm. & Hoehne (Fabaceae)	0.00	0.01	1	Lauraceae sp.1	1	0.04
189	*Miconia minutiflora* (Bonpl.) DC. (Melastomataceae)	0.00	0.01	1	Lauraceae sp.2	1	0.04
190	*Jacaratia heptaphylla* (Vell.) A. DC. (Caricaceae)	0.00	0.01	1	*Lonchocarpus cultratus* (Vell.) A.M.G. Azevedo & H.C. Lima (Fabaceae)	1	0.04
191	Lauraceae sp.1	0.00	0.01	1	*Machaerium caratinganum* Kuhlm. & Hoehne (Fabaceae)	1	0.04
192	Euphorbiaceae sp.2	0.00	0.01	2	*Machaerium* sp. (Fabaceae)	1	0.04
193	*Piper cernuum* Vell. (Piperaceae)	0.00	0.01	2	*Marlierea suaveolens* Cambess. (Myrtaceae)	1	0.04
194	*Stylogyne pauciflora* Mez (Primulaceae)	0.00	0.01	1	*Maytenus floribunda* Reissek (Celastraceae)	1	0.04
195	*Erythroxylum pelleterianum* A. St.-Hil. (Erythroxylaceae)	0.00	0.01	1	*Miconia brunnea* Mart. ex DC. (Melastomataceae)	1	0.04
196	Lauraceae sp.2	0.00	0.01	1	*Miconia cinnamomifolia* (DC.) Naudin (Melastomataceae)	1	0.04
197	*Psychotria myriantha* Müll. Arg. (Rubiaceae)	0.00	0.01	1	*Miconia minutiflora* (Bonpl.) DC. (Melastomataceae)	1	0.04
198	Rubiaceae sp.	0.00	0.01	2	*Miconia tristis* Spring (Melastomataceae)	1	0.04
199	*Casearia decandra* Jacq. (Salicaceae)	0.00	0.01	1	*Naucleopsis oblongifolia* (Kuhlm.) Carauta (Moraceae)	1	0.04
200	*Maytenus floribunda* Reissek (Celastraceae)	0.00	0.01	1	*Ocotea pulchella* (Nees & Mart.) Mez (Lauraceae)	1	0.04
201	*Terminalia glabrescens* Mart. (Combretaceae)	0.00	0.01	1	Oleaceae sp.	1	0.04
202	*Brunfelsia uniflora* (Pohl) D. Don (Solanaceae)	0.00	0.01	1	*Plinia grandifolia* (Mattos) Sobral (Myrtaceae)	1	0.04
203	Euphorbiaceae sp.1	0.00	0.01	1	*Prockia crucis* P. Browne ex L. (Salicaceae)	1	0.04
204	*Randia ferox* (Cham. & Schltdl.) DC. (Rubiaceae)	0.00	0.01	2	*Prunus sellowii* Koehne (Rosaceae)	1	0.04
205	*Psidium oblongatum* O. Berg (Myrtaceae)	0.00	0.01	1	*Psidium oblongatum* O. Berg (Myrtaceae)	1	0.04
206	Solanaceae sp.	0.00	0.01	1	*Psychotria myriantha* Müll. Arg. (Rubiaceae)	1	0.04
207	*Celtis iguanaea* (Jacq.) Sarg. (Cannabaceae)	0.00	0.00	1	*Psychotria rhytidocarpa* Müll. Arg. (Rubiaceae)	1	0.04
208	*Miconia tristis* Spring (Melastomataceae)	0.00	0.00	1	*Psychotria vellosiana* Benth. (Rubiaceae)	1	0.04
209	Euphorbiaceae sp.3	0.00	0.00	1	*Qualea multiflora* Mart. (Vochysiaceae)	1	0.04
210	*Psychotria* sp. (Rubiaceae)	0.00	0.00	2	*Sloanea hirsuta* (Schott) Planch. ex Benth. (Elaeocarpaceae)	1	0.04
211	*Vantanea obovata* (Nees & Mart.) Benth. (Humiriaceae)	0.00	0.00	1	Solanaceae sp.	1	0.04
212	*Handroanthus chrysotrichus* (Mart. ex A. DC.) Mattos (Bignoniaceae)	0.00	0.00	1	*Stylogyne pauciflora* Mez (Primulaceae)	1	0.04
213	*Machaerium* sp. (Fabaceae)	0.00	0.00	1	*Swartzia acutifolia* Vogel (Fabaceae)	1	0.04
214	*Cybianthus fuscus* Mart. (Primulaceae)	0.00	0.00	1	*Terminalia glabrescens* Mart. (Combretaceae)	1	0.04
215	Guatteria sp.2 (Annonaceae)	0.00	0.00	1	Unidentified sp.3	1	0.04
216	*Psychotria rhytidocarpa* Müll. Arg. (Rubiaceae)	0.00	0.00	1	Unidentified sp.4	1	0.04
217	*Oleaceae* sp.	0.00	0.00	1	*Vantanea obovata* (Nees & Mart.) Benth. (Humiriaceae)	1	0.04
218	*Psychotria vellosiana* Benth. (Rubiaceae)	0.00	0.00	1	*Vernonia diffusa* Less. (Asteraceae)	1	0.04

**Table 6.** Description of the Darwin Core Archive **dwca-2015-06-04-gbif-ipt-unesco-ufv-seunico-fsn.zip** (Suppl. material 2​)

**file**	**Description**
occurrence.txt	core occurrence file, contains a list of occurrences (individuals) in this dataset
meta.xml	(DwC-) archive descriptor
measurementorfactplots.txt	extension file: measurements or facts, description of the chemical soil properties and understory light availability in plots where occurrences were registered (due to technical issues, extension files measurementorfactplots.txt and measurementorfactplants.txt were merged to the single extension file measurementorfact.txt)
measurementorfactplants.txt	extensionfile: height, diameter at breast height as well as basal area of all occurrences during both census (due to technical issues, extension files measurementorfactplots.txt and measurementorfactplants.txt were merged to the single extension file measurementorfact.txt)
description.txt	extension file: habitat, contains habitat type in which the taxon was registered
eml.xml	meta data document
resourcerelationship.txt	extension file: describes the relationship among occurrences and plots

**Table 7.** Environmental data from 100 subplots from the FSN Dynamics Plot.

**Variable**	**Mean ±SD**	**Minimum**	**Maximum**
**Understory light availability**			
Canopy Openness [%]	4.56 ±1.34	1.81	8.07
Effective leaf area index integrated over the zenith angles 0 to 60º	3.85 ±0.57	2.93	6.16
Effective leaf area index integrated over the zenith angles 0 to 75º	3.65 ±0.51	2.77	5.43
Absolut amount of direct (bean) radiation found below the canopy and the topographic mask [mol/m^2^/d]	1.82 ±0.63	0.38	3.81
Absolut amount of diffuse radiation found below the canopy and the topographic mask [mol/m^2^/d]	1.37 ±0.38	0.62	2.40
Absolut amount of total radiation found below the canopy and the topographic mask [mol/m^2^/d]	3.19 ±0.97	1.01	6.20
**Soil properties**			
Soil acidity (pH)	4.43 ±0.32	3.96	5.63
Sum of interchangeable bases [cmol_c_/dm^3^]	1.15 ±1.07	0.14	6.39
Effective cation exchange capacity [cmol_c_/dm^3^]	2.67 ±0.59	1.72	6.39
Saturation of bases []	10.96 ±9.7	1.27	51.43
Potential soil acidity [cmol_c_/dm^3^]	9.25 ±1.5	6.03	13.30
Phoshprous availability [mg/dm^3^]	2.38 ±0.62	1.43	4.03
Aluminium saturation [%]	60.9 ±26.54	0.00	95.10
Aluminium availability [cmol_c_/dm^3^]	1.52 ±0.68	0.00	2.89
Remaining phosphorus []	26.4 ±3.2	18.40	33.63
Potassium availability [mg/dm^3^]	62.31 ±32.2	24.00	231.00
Organic matter [dag/kg]	5.26 ±0.72	3.50	7.52
Cation exchange capacity at pH 7 [cmol_c_/dm^3^]	10.4 ±1.16	8.08	13.61
Magnesium availability [cmol_c_/dm^3^]	0.41 ±0.37	0.04	2.14
Calcium availability [cmol_c_/dm^3^]	0.58 ±0.63	0.00	3.66
Nitrogen availability [dag/kg]	0.25 ±0.03	0.19	0.32

**Table 8.** Canopy openness and total understory radiation (mean and standard deviations) in northern and southern subplots from the FSN dynsamics plot. P is significance level of difference according to a two-tailed t-test.

**Variable**	**Northern subplots**	**Southern subplots**	**P**
Canopy openness [%]	4.13 ±1.21	4.83 ±1.35	< 0.01
Absolut amount of total radiation found below the canopy and the topographic mask [mol/m^2^/d]	2.86 ±0.78	3.40 ±1.02	< 0.005

**Table 9.** Column labels and descriptions of measurementorfactplots.txt from Darwin Core Archives **dwca-2015-06-04-gbif-ipt-unesco-ufv-seunico-fsn.zip** (Suppl. material 2​) conntaining soil properties and understory light availability related to tree occurrences.

**Column label**	**Column description**
Id	Occurrence identifier
measurementType	Description of the measurement, these are pH; phosphorus (P), potassium (K), aluminium (Al), calcium (Ca) and magnesium (Mg) availability; potential acidity; saturation of bases and of aluminium; effective cation exchange capacity; cation exchange capacity at pH 7.0; interchangeable bases; remaining phosphorus; absolute amount of diffuse, direct and total radiation found below the canopy and the topographic mask as well as canopy openness
measurementValue	Value of the measurement
measurementUnit	Units associated with the measurementValue
measurementDeterminedDate	Date on which the measurement was carried out
measurementMethod	Description of the method used to determine plot properties
measurementRemarks	Comments or notes accompanying the measurement

**Table 10.** Column labels and descriptions of measurementorfactplants.txt from Darwin Core Archives **dwca-2015-06-04-gbif-ipt-unesco-ufv-seunico-fsn.zip** (Suppl. material 2​) containing basal area, height and diameter measures of tree occurrences

**Column label**	**Column description**
Id	Occurrence identifier
measurementType	Description of the measurement, this is diameter at breast height, basal area and height
measurementValue	Value of the measurement
measurementUnit	Unit of measurement
measurementDeterminedDate	Date on which the measurement was carried out
measurementMethod	Description of the applied method (measurement or estimation)
measurementRemarks	Comments or notes accompanying the measurement

**Table 11.** Column labels and descriptions of resourcerelationship.txt from Darwin Core Archives **dwca-2015-06-04-gbif-ipt-unesco-ufv-seunico-fsn.zip** (Suppl. material 2​) describing resource relationships among occurrences and plots

**Column label**	**Column description**
Id	Occurrence identifier
relatedResourceID	Identifier for related resource
relationshipOfResource	The relationship of the resource identified by relatedResourceID to the tree occurrence
relationshipRemark	Further comments or notes about the relationship among tree occurrences and plots

**Table 12.** Column labels and descriptions of description.txt from Darwin Core Archives **dwca-2015-06-04-gbif-ipt-unesco-ufv-seunico-fsn.zip** (Suppl. material 2​​)

**Column label**	**Column description**
Id	Occurrence identifier
description	Habitat type at location where occurrence was registered, i.e., seasonal semideciuous forest
type	The kind of description
language	The language of the ressource
